# The role of airway macrophages in apoptotic cell clearance following acute and chronic lung inflammation

**DOI:** 10.1007/s00281-016-0555-3

**Published:** 2016-03-08

**Authors:** Aleksander M. Grabiec, Tracy Hussell

**Affiliations:** Manchester Collaborative Centre for Inflammation Research, Core Technology Facility, The University of Manchester, 46 Grafton Street, M13 9NT Manchester, UK

**Keywords:** Efferocytosis, Phosphatidylserine, Apoptotic cell clearance, Lung, Airway macrophage, Inflammation

## Abstract

Acute and chronic inflammatory responses in the lung are associated with the accumulation of large quantities of immune and structural cells undergoing apoptosis, which need to be engulfed by phagocytes in a process called ‘efferocytosis’. Apoptotic cell recognition and removal from the lung is mediated predominantly by airway macrophages, though immature dendritic cells and non-professional phagocytes, such as epithelial cells and mesenchymal cells, can also display this function. Efficient clearance of apoptotic cells from the airways is essential for successful resolution of inflammation and the return to lung homeostasis. Disruption of this process leads to secondary necrosis of accumulating apoptotic cells, release of necrotic cell debris and subsequent uncontrolled inflammatory activation of the innate immune system by the released ‘damage associated molecular patterns’ (DAMPS). To control the duration of the immune response and prevent autoimmune reactions, anti-inflammatory signalling cascades are initiated in the phagocyte upon apoptotic cell uptake, mediated by a range of receptors that recognise specific phospholipids or proteins externalised on, or secreted by, the apoptotic cell. However, prolonged activation of apoptotic cell recognition receptors, such as the family of receptor tyrosine kinases Tyro3, Axl and MerTK (TAM), may delay or prevent inflammatory responses to subsequent infections. In this review, we will discuss recent advances in our understanding of the mechanism controlling apoptotic cell recognition and removal from the lung in homeostasis and during inflammation, the contribution of defective efferocytosis to chronic inflammatory lung diseases, such as chronic obstructive pulmonary disease, asthma and cystic fibrosis, and implications of the signals triggered by apoptotic cells in the susceptibility to pulmonary microbial infections.

Cellular turnover occurs as part of the normal homeostatic process and is vital for the contraction of cells recruited during an inflammatory response [1]. As with most biological processes, it is becoming apparent that turnover of cells is regulated in a tissue-specific manner and this specificity is primarily driven by locally produced factors. For example, the unique microenvironment of the airspaces drives a distinct expression pattern of apoptotic cell recognition receptors on airway macrophages compared to tissue-resident macrophages from other anatomical locations [2]. In the airways, cell turnover is primarily mediated by induction of apoptosis followed by engulfment of apoptotic cells by phagocytes, called ‘efferocytosis’. The term ‘efferocytosis’ was coined to separate engulfment of apoptotic cells from other forms of phagocytosis and derived from the Latin word ‘effere’ that translates as ‘take to the grave’. While phagocytosis of pathogens requires initiation of an inflammatory response, removal of self-cells that undergo apoptosis is a tolerogenic process that prevents excessive inflammation and/or autoimmunity caused by the release of ‘damage associated molecular patterns’ (DAMPS) from apoptotic cells that are not cleared in a timely manner and undergo secondary necrosis. To achieve this distinction, macrophages and other phagocytes utilise different sets of receptors to recognise microbial particles and cells undergoing apoptosis, activation of which subsequently leads to distinct cell activation programmes. Whereas phagocytosis of pathogens triggers conserved pro-inflammatory signalling pathways, engulfment of apoptotic cells by phagocytes may promote the wound repair process, the production of factors to curtail inflammation or the secretion of growth factors to shape a developing tissue. Efferocytosis therefore leads to different consequences and is a burgeoning focus of research, driven by the discovery of ever more apoptotic cell recognition receptors and, importantly, pathogens that use the apoptotic cell clearance process to facilitate their entry into host cells and/or subvert anti-pathogen immunity.

## Mechanisms of efferocytosis

The distinction between apoptotic and healthy cells is mediated primarily by the release of ‘find-me’ signals from apoptotic cells that include ATP and UTP [3], fractalkine (CX3CL1) [4], lysophosphatidylcholine [5] and sphingosine-1-phosphate [6]. In addition to recruitment, such soluble signals may also enhance the engulfment capacity of the responding cell. For example, apoptotic cell production of fractalkine induces milk fat globule-epidermal growth factor 8 (MFG-E8) expression in phagocytes that enhances apoptotic cell clearance [7].

Apoptotic cells expose a variety of molecules on their cell surface that can be recognised by receptors on phagocytic cells. The precise composition of such ‘eat-me’ signals likely depends on whether apoptosis is occurring under homeostatic conditions or during inflammation and is tissue- and cell type-specific [1, 8]. One of the best studied ‘eat-me’ signals is phosphatidylserine (PtdSer) that in living cells is localised to the inner leaflet of the plasma membrane and is externalised upon induction of apoptosis [9]. Overexpression of PtdSer enhances apoptotic cell engulfment, whereas PtdSer blockade suppresses this process, leading to autoimmunity [10, 11]. Irreversible externalisation occurs when caspases inactivate flippase (ATP11C) that in healthy cells continually flips PtdSer in the plasma membrane [12]. At the same time, caspase-dependent activation of scramblases (such as Xkr8), which non-specifically and bi-directionally scramble phospholipids in the plasma membrane, is also required for PtdSer exposure on apoptotic cells [13]. PtdSer is transiently exposed on the surface of living cells that resist efferocytosis, suggesting that this signal alone may not be enough, or that prolonged interaction of PtdSer receptors on phagocytes is required [14]. Oxidised low-density lipoprotein, calreticulin, annexin A1, ICAM3, C1q and thrombospondin-1 (TSP1) are also implicated in efferocytosis [1]. Furthermore, healthy cells prevent engulfment by expressing CD47 and CD31 [15, 16] or by ligating CD300a that impedes macrophage function [17]. A balance of efferocytosis-inducing and inhibitory signals may therefore determine apoptotic cell clearance.

PtdSer and other ‘eat-me’ signals exposed on apoptotic cells bind a plethora of receptors on phagocytic cells, a number of which have been identified in the lung (Table 1). Externalised PtdSer is recognised directly by triggering receptor expressed by myeloid cells-2 (TREM2) [30], CD300 [31], receptor for advanced glycation end products (RAGE) [32], stabilin-2 [33], brain-specific angiogenesis inhibitor-1 (BAI1) [34] and the family of T cell/transmembrane, immunoglobulin, and mucin (TIM) receptors, which includes TIM-1, 3 and 4 [35, 36]. Other engulfment receptors require bridging molecules to link them to externalised PtdSer. For example, integrin αvβ3 or αvβ5 requires MFG-E8, while Tyro3, Axl and MerTK that form the TAM receptor tyrosine kinase family [37] require the bridging molecules Protein S or growth arrest specific 6 (Gas6) [38]. The N-terminal Gla domains of Protein S and Gas6 bridge TAM receptors to PtdSer on the surface of apoptotic cells [39], whereas the C-terminal sex hormone-binding globulin-like domains bind and activate the TAM receptor [40]. There are also a number of receptors that recognise other externalised ligands including scavenger receptor class F, member 1 (SCARF1) and LDL receptor-related protein-1 (LRP-1, also known as CD91) that recognise C1q and calreticulin, respectively, CD36 together with integrin αvβ3 or αvβ5 that recognises TSP-1, CD14 that binds a modified form of the intracellular adhesion molecule ICAM3, and lectin receptors that recognise altered sugars [1].

**Table 1:** ‘Eat-me’ signals exposed on apoptotic cells and their recognition receptors expressed on phagocytes

‘Eat-me’ signal	Receptor	Receptor expression on airway macrophages
PtdSer	TIM family (TIM-1, 3 and 4)	Not studied
	TAM family (Tyro3, Axl, MerTK) through Gas6 and Protein S	High Axl and MerTK expression in mice, only MerTK studied and confirmed in humans [2, 18]
	BAI1	Not studied
	RAGE	Confirmed in mice [19] and humans [20]
	Stabilin-2	Not studied
	Integrin αvβ3 or αvβ5 through MFG-E8	Low αvβ3 expression in humans [21, 22]
	TREM2	Confirmed in mice [23] and humans [24]
TSP-1	CD36 (in complex with integrin αvβ3 or αvβ5)	Confirmed in humans [21, 25]
ICAM3	CD14	Low expression in mice [26] and humans [27]
Altered sugars	Lectin receptors	Mannose receptor confirmed in mice [26] and humans [28]
Calreticulin	LRP-1/CD91	Confirmed in mice [29] and humans [22, 29]
C1q	SCARF1	Not studied

*BAI1* brain-specific angiogenesis inhibitor-1, *ICAM3* intracellular adhesion molecule-3, *LRP-1* LDL receptor-related protein-1, *MFG-E8* milk fat globule-epidermal growth factor 8, *PtdSer* phosphatidylserine, *RAGE* receptor for advanced glycation end products, *TSP-1* thrombospondin-1, *SCARF1* scavenger receptor class F, member 1, *TIM* T cell/transmembrane, immunoglobulin, and mucin, *TREM2* triggering receptor expressed on myeloid cells-2

The logic behind possessing so many receptors that can recognise apoptotic cells is not entirely clear. Some, such as TIM-4, act as tethering receptors without any signalling consequences [41], similar to CD14 [42]. Different receptors may also act at different stages of efferocytosis [43] or may preferentially clear cells in different locations. For example, TREM2 and TREM2-L form a receptor-ligand pair connecting microglia with apoptotic neurons, directing removal of damaged cells to allow repair [44]. It is also likely that an alternate outcome is required upon efferocytosis that requires linkage to different signalling components [31]. With regard to the TAM receptors, MerTK is ubiquitously expressed on macrophages and even used as a defining marker for them. Airway macrophages, however, unlike most other macrophages, constitutively express Axl, possibly due to the local environment that is rich in granulocyte-macrophage colony-stimulating factor (GM- CSF) [2]. Importantly, receptors that recognise apoptotic cells can also play a dual function: inducing the cytoskeletal rearrangements necessary to ingest the apoptotic cell and also transmitting an instructive signal [45]. It is interesting to note that individual TAM receptor family members use different molecules to bridge them to PtdSer externalised on apoptotic cells: MerTK and Tyro3 are activated by both Gas6 and Protein S, whereas the sole ligand for Axl is Gas6 [46, 47]. In the case of MerTK and Tyro3, it is therefore possible that specific signals triggered by receptor ligation might differ depending on the bridging molecule, though this possibility remains to be verified experimentally. Finally, further selectivity of response is afforded by co-operation of multiple receptors such as Axl and LRP-1 on dendritic cells where Axl tethers the apoptotic cell to dendritic cells, but LRP-1 is required to trigger internalisation [48].

## Impact of efferocytosis on cell function

**Fig. 1:**
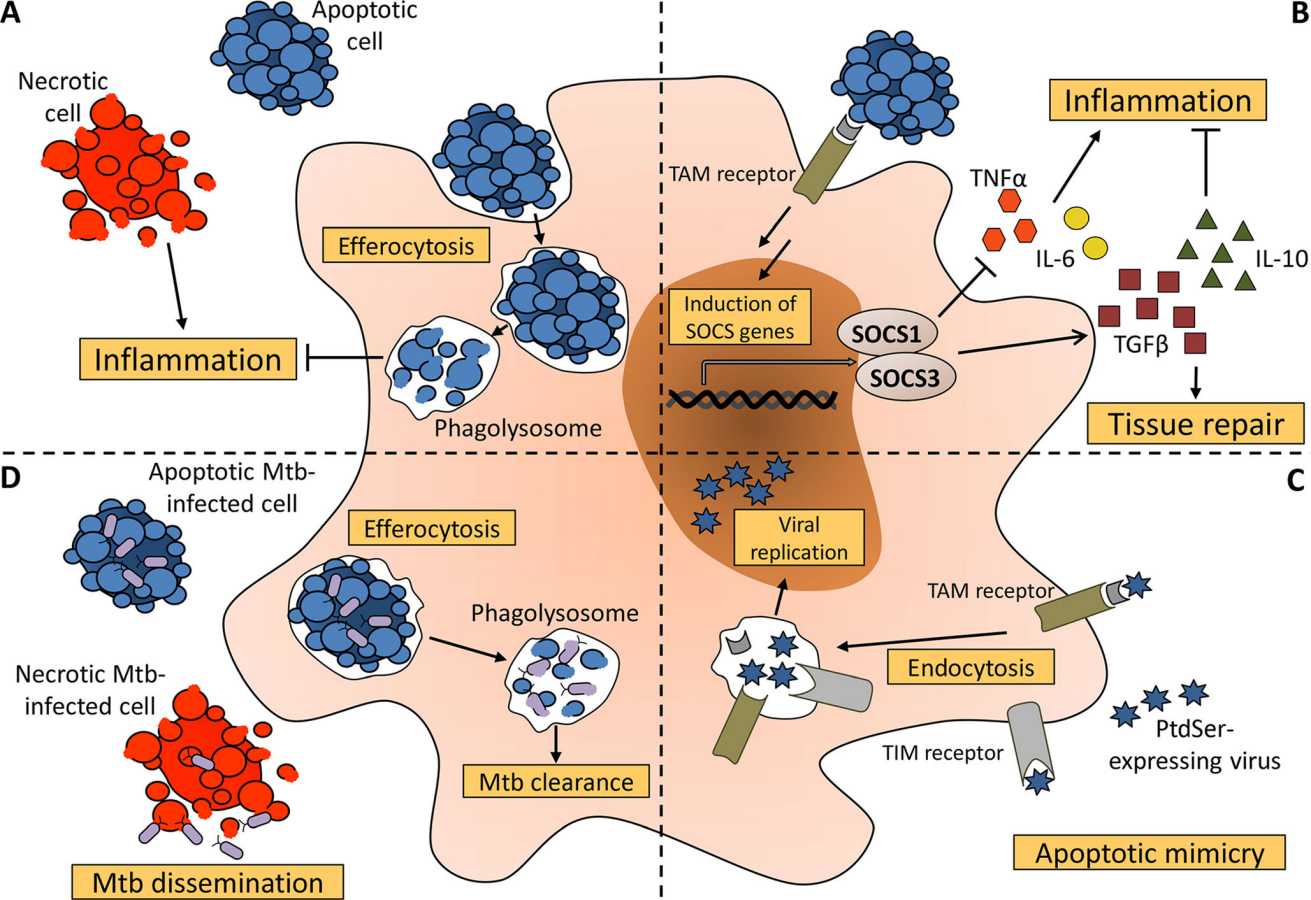
The many roles of apoptotic cell recognition and phagocytosis in immunity and infection. **a** Efferocytosis leads to removal of apoptotic cells without release of their content. When apoptotic cells are not engulfed in a timely manner, they undergo secondary necrosis and release necrotic cell debris which subsequently causes uncontrolled inflammatory activation of the innate immune system by the released ‘damage associated molecular patterns’ (*DAMPS*). **b** During apoptosis, cells expose phosphatidylserine (*PtdSer*) on the outer leaflet of their membranes, which is recognised by specific receptors expressed on phagocytes. Recognition of PtdSer by TAM receptors through bridging molecules Gas6 and Protein S triggers a signalling cascade which converges on upregulation of suppressor of cytokine signalling-1 and 3 (SOCS3), which act as negative regulators of the immune response. Activation of TAM receptors by apoptotic cells inhibits production of proinflammatory cytokines, such as TNFα and IL-6, while promoting expression of factors that suppress inflammation and promote tissue repair, including IL-10 and TGF-β. **c** Some enveloped viruses express PtdSer on their envelopes and use PtdSer recognition receptors, such as TAM and TIM receptor families, to promote infection of the host cells and evade the immune response. **d** During microbial infections with intracellular pathogens, induction of apoptosis of infected cells is one of the strategies of the host immune system to facilitate pathogen clearance. For example, in case of *Mycobacterium tuberculosis* (*Mtb*) infections, necrosis of infected cells leads to dissemination of bacteria, whereas engulfment of infected cells undergoing apoptosis allows for pathogen destruction

The receptors that mediate efferocytosis often have anti-inflammatory signalling consequences that can change the phenotype and function of the ingesting cell. For example, engagement and activation of TAM receptors inhibits signalling pathways triggered by cytokines and toll-like receptor ligands through induction of suppressor of cytokine signalling-1 and 3 (SOCS-1 and 3) [49, 50] (see Fig. 1a, b). The impact of apoptotic cell clearance on cell function depends on the cell type mediating efferocytosis, which in turn depends on tissue location. In the lung, efferocytosis is mediated predominantly by macrophages and airway epithelial cells, with most consequences studied in the former. In macrophages, efferocytosis increases the secretion of the anti-inflammatory cytokines, transforming growth factor-β (TGF-β) and interleukin (IL)-10 [51, 52], while inhibiting secretion of proinflammatory mediators such as TNF-α, IL-1, IL-8 and leukotriene C4 [53, 54]. IL-10 production by macrophages upon apoptotic cell contact is, in part, dependent upon the scavenger receptor CD36 [51] and TSP-1, which links macrophages to apoptotic cells in cooperation with integrin αvβ3 [55]. Efferocytosis also upregulates prostaglandin E2 (PGE2) and impairs FcR-mediated phagocytosis [54, 56], and intratracheal instillation of apoptotic cells enhances the resolution of LPS-induced acute pulmonary inflammation [57]. This change in function from pro-inflammatory to pro-resolution can be observed as a phenotypic switch from an M1- to an M2-like macrophage phenotype and includes the induction of peroxisome proliferator-activated receptor-γ (PPARγ) [58]. However, this is likely to be context-, cell-type- and tissue-dependent. For example, nitric oxide, but not IL-10, TGF-β or PGE2, mediates the immunosuppressive effects induced by apoptotic cell efferocytosis in dendritic cells [59]. It is also interesting to note that efferocytosis by dendritic cells appears to be subset-specific. Only CD103^+^ murine lung dendritic cells capture and present apoptotic cell-associated antigen in health and disease, whereas both the CD11b^hi^ and the CD103^+^ dendritic cells ingest and traffic latex beads or soluble antigen [60]. Whether this is due to selective expression of receptors recognising apoptotic cells or a phenotypic switch after ingestion is not currently known. Therefore, clearance of apoptotic cells has profound influences on the ingesting cell. Though the outcome has predominantly been studied for macrophages, other cell types, particularly dendritic cells, are also likely to be affected.

## Cell turnover and clearance in the lung

In any tissue, cell turnover is a natural homeostatic process and occurs predominantly by apoptosis before loss of plasma membrane integrity. Timely removal of these apoptotic cells is critical to prevent autoimmune reactions to cellular constituents. However, the speed of turnover determines the requirements for the local phagocytic system in terms of its capacity to clear the accumulating apoptotic cells and is often governed by the local microenvironment and past inflammation history. In healthy murine and human lungs, airway macrophages make up 95 % of cells retrieved by bronchoalveolar lavage [2, 61]. They exist in a tolerant state due to interaction with proteins and receptors expressed by or secreted from the respiratory epithelium. Furthermore, mucus bathing the epithelium contains surfactant proteins that dampen macrophage responsiveness in health [62]. Chimeric mouse experiments show that resident airway macrophages have an unusually prolonged life span with negligible turnover even at 8 months in the absence of inflammation. This situation changes following their depletion during inflammation where replenishment from the periphery occurs, leading to a population of macrophages that are reported to have a shorter life span. Airway macrophage removal by irradiation leads to replenishment from the periphery and a higher turnover rate of approximately 30 days [63]. However, the fate of resident airway macrophages following pulmonary infection is not entirely clear. One study shows that macrophages recruited to the airways from the periphery during influenza infection contract by Fas-dependent apoptosis, while the number of resident airway macrophages remains unchanged [64], whereas another implies significant ablation of resident airway macrophages followed by replenishment from the interstitial pool [65]. Discrepancy might arise from the use of intranasal dye (that becomes diluted on cell division) or the depletion by irradiation with the lung protected by lead. In order to track definitively the fate of airway macrophages following influenza infection, the Flt3-Cre × Rosa26-LSL-YFP reporter mice would be useful as all bone marrow emigrants become YFP expressing and so mice do not require prior manipulation to distinguish resident versus recruited airway cells [66]. Like airway macrophages, epithelial cells similarly have a long half-life in health. Using reporter mice where ciliated cells and their progeny can be tracked across their lifetime, a half-life of 6 months in the trachea and 17 months in the bronchioles and terminal bronchioles has been estimated [67].

Taken together, these observations indicate that in homeostasis relatively small quantities of apoptotic cells appear in the airways due to slow turnover of the main cell populations residing in the healthy lung. It is therefore not surprising that lung phagocytes efficiently clear these scarce apoptotic cells even when their efferocytic function is partly disrupted. For example, deletion of Axl, which is highly expressed on murine airway macrophages, does not cause accumulation of necrotic cell debris and lung inflammation in the absence of accompanying infection despite significant impairment of the efferocytic function of airway macrophages [2]. Similarly, blockade of apoptotic cell uptake by airway epithelial cells through cell type-specific deletion of the small GTPase Rac1, which is required for efferocytosis mediated by several classes of engulfment receptors, does not affect the integrity and responses of the epithelial barrier without administration of exogenous apoptotic cells or induction of allergic airway inflammation [68].

## Defective efferocytosis in chronic lung diseases

In health, apoptotic cells are essentially undetectable in the lung tissue [69], and immune cells infiltrating the airways which undergo apoptosis are rapidly cleared by airway macrophages in models of acute microbial infection-related lung inflammation [70]. Respiratory infections are therefore not associated with persistent accumulation of large quantities of apoptotic cells in the airspaces. However, increased numbers of dying cells are detected in the airspaces of patients with several aetiologically distinct chronic inflammatory lung diseases [71]. Although higher frequencies of apoptotic cells in these diseases might result either from elevated rates of apoptotic cell death or defects in apoptotic cell removal, experimental evidence suggests that reduced efficiency of efferocytosis, predominantly by airway macrophages, plays a key role in this pathological process [72, 73].

### Chronic obstructive pulmonary disease

The most comprehensive analysis of defects in apoptotic cell clearance has been performed in chronic obstructive pulmonary disease (COPD), which is a chronic lung disease characterised by un-resolving inflammation due to accumulation of activated neutrophils and T cells causing small-airway obstruction, peribronchial fibrosis and/or destruction of airway walls (emphysema). These pathological processes lead to progressive destruction of lung parenchyma and, as a consequence, airflow limitation that is not fully reversible in patients with emphysema, and in other subtypes of COPD [74]. Historically, lung pathology in COPD was attributed to alterations in the protease/anti-protease balance and oxidative stress caused by cigarette smoking, which is the main risk factor for COPD. However, there is evidence that indicates an important role of aberrant apoptosis and apoptotic cell clearance in the lung damage observed in COPD [75]. Several reports demonstrate accumulation of apoptotic epithelial, endothelial and immune cells in the lungs of patients with COPD and/or emphysema [69, 76, 77], and induction of structural airway cell apoptosis is sufficient to cause emphysematous changes in mice [78].

In a series of elegant studies, Hodge and co-workers demonstrate that increases in the numbers of apoptotic cells detected in the lungs of patients with COPD can be attributed to significantly impaired efferocytic function of airway macrophages. Uptake of apoptotic bronchial epithelial cells and neutrophils is significantly reduced in bronchoalveolar lavage macrophages from patients with COPD compared to healthy controls [21, 22, 79], and this defect is more pronounced in COPD patients who currently smoke [22]. The observed reductions in efferocytic potential of airway macrophages in vitro correlate with the frequency of apoptotic bronchial epithelial cells isolated from bronchial brushings and are associated with altered expression of several proteins involved in apoptotic cell recognition and binding, including CD31, CD44 and LRP-1/CD91 [22]. Intriguingly, restoration of COPD airway macrophage efferocytic function by the macrolide antibiotic azithromycin does not correlate with changes in expression of these molecules [21]. Instead, it depends on PtdSer binding by airway macrophages [21], suggesting that deregulation of PtdSer-recognising receptors or bridging molecules might be responsible for defective apoptotic cell removal in COPD. Neither TAM nor TIM families of PtdSer recognition receptors have been studied in COPD so far but, surprisingly, increased expression of MerTK is observed on airway macrophages from cigarette smokers [18], which are also characterised by impaired apoptotic cell uptake compared to cells from healthy non-smokers [18, 22]. This observation indicates that upregulation of MerTK is not sufficient to restore the efforocytic function of airway macrophages to normal levels and other PtdSer receptors, such as Axl, which is highly expressed on mouse airway macrophages [2], might play a more prominent role.

Apart from potential alterations in PtdSer receptor expression and function, which require more detailed studies, several other mechanisms might be involved in efferocytosis defects observed in COPD. Significant impairment of apoptotic cell uptake by airway macrophages from smokers indicates an important role of oxidative stress caused by cigarette smoke components in this process [18, 22]. In mice, cigarette smoke exposure, which is sufficient to induce emphysema [80], significantly suppresses efferocytosis by airway macrophages in vivo and in vitro, and this effect is reversible by treatment with antioxidants or overexpression of extracellular superoxide dismutase [81, 82]. Macrophage phagocytosis of apoptotic cells is also inhibited by the alarmin high mobility group protein-1 (HMGB1) [83, 84], and elevated levels of HMGB1 both in the airways and peripheral blood of patients with COPD have recently been reported, negatively correlating with patient lung function [20, 85]. Since necrotic cells are the main source of extracellular HMGB1, a positive feedback loop might exist in COPD where apoptotic cells undergo secondary necrosis and release HMGB1, which further impairs the efferocytic function of airway macrophages. This in turn would lead to accumulation of greater amounts of necrotic debris and perpetuation of chronic inflammation. Finally, engulfment of apoptotic cells by macrophages is also regulated by the members of the collectin family of C-type lectins. The levels of mannose-binding lectin (MBL), which promotes apoptotic cell uptake in vitro [86], are reduced in the airways of patients with COPD and correlate with impaired macrophage efferocytosis [87, 88]. Surfactant protein A (SP-A) and SP-D, on the other hand, play a more complex role in apoptotic cell recognition in the lung. While homeostatic interaction of SP-A or SP-D with signal inhibitory regulatory protein-α (SIRPα) expressed on airway macrophages suppresses efferocytosis [89], opsonisation of apoptotic cells by SP-A or SP-D and interaction of this complex with LRP-1/CD91 enhances apoptotic cell uptake [29, 90]. The latter seems to play a more prominent role during chronic lung inflammation as SP-D levels are significantly reduced in patients with COPD [28, 91], potentially contributing to efferocytosis defects. Alternatively, downregulation of SP-D might represent a physiological response of the lung aimed at overcoming SIRPα-dependent suppression of macrophage efferocytic function in the presence of large amounts of dead cells, which fail to efficiently remove apoptotic cells from the COPD airways due to other phagocytosis defects.

### Asthma

Asthma is an obstructive airway disease characterised by hyper-responsiveness and chronic inflammation of the respiratory tract. While neutrophils are a predominant cell type in the lungs of COPD patients, asthma represents eosinophil-dominant airway inflammation. Initial priming of the adaptive immune system by airway allergens leads to the activation of tissue-resident mast cells, which release a broad array of inflammatory mediators promoting migration of eosinophils into the airways [92]. Products of eosinophil degranulation, including major basic protein, eosinophil cationic protein and reactive oxygen species, are cytotoxic to epithelial cells and cause airway damage and tissue remodelling. Indeed, increased numbers of apoptotic epithelial cells have been detected in the lungs of patients with asthma compared to healthy individuals [93]. Apoptosis and subsequent uptake by phagocytes appear to be a predominant mechanism of eosinophil removal from the airways [94, 95], and glucocorticoids, which are commonly used to control inflammation in asthma, induce eosinophil apoptosis [96]. These observations indicate that high efficiency of the phagocytic system is required for removal of large quantities of apoptotic eosinophils and bronchial epithelial cells from the airways of patients with asthma and raise the possibility that defects in efferocytosis might prevent resolution of lung inflammation.

Initial studies by Hyunh et al. demonstrate that airway macrophages from patients with severe asthma contain reduced numbers of phagocytic bodies compared to healthy donors and patients with mild/moderate asthma and are defective in phagocytosing apoptotic Jurkat T cells in vitro [97]. Failure of airway macrophages from patients with severe asthma to efficiently clear apoptotic cells is associated with reduced production of anti-inflammatory eicosanoids [97], suggesting that defects in efferocytosis might contribute to perpetuation of inflammation in asthma, not only through secondary necrosis of apoptotic cells, but also by reduced release of regulatory mediators that are normally produced upon recognition of apoptotic cells by airway macrophages. More recently, it has been shown that efferocytosis of apoptotic bronchial epithelial cells by airway macrophages isolated from induced sputum of patients with non-eosinophilic asthma is significantly reduced compared with patients with eosinophilic asthma, and the degree of uptake impairment is comparable to that observed in COPD [98]. This observation might partly explain the persistent neutrophilia and aberrant innate immune responses in this subgroup of patients [99]. Efferocytosis defects in asthma are associated not only with the subtype of airway inflammation but also with the BMI index: airway macrophages from obese patients with asthma, which typically suffer from more severe disease symptoms [100], are characterised by significantly reduced numbers of phagocytic bodies compared to non-obese asthmatics [101]. Notably, defective efferocytic function in obese asthmatics is not restricted to the site of inflammation as decreased uptake of beads mimicking apoptotic cells is also observed in peripheral blood monocytes isolated from these patients, correlating with reduced glucocorticoid responsiveness [101].

While these reports provide clear evidence that removal of apoptotic cells is defective in asthma, in particular in certain disease subtypes, additional studies are needed to characterise the molecular mechanisms underlying the observed defects. Even though potential changes in expression and function of receptors recognising apoptotic cells on airway macrophages have not been formally studied in asthma, it is important to note that the TIM receptor family member TIM-1, which is preferentially expressed on specific lymphocyte populations, is an important susceptibility gene for allergic asthma [102]. Targeting TIM-1 modulates airway inflammation in mouse models of airway hyper-responsiveness at least in part through TIM-1-dependent interaction of NKT cells with PtdSer on the surface of apoptotic cells [103, 104]. These observations suggest that recognition of PtdSer by immune cells that do not phagocytose apoptotic cells also has profound effects on the development of chronic lung inflammation. They also indicate that future studies of molecules involved in apoptotic cell binding in asthma should not be restricted to analyses of airway macrophages as the main contributors to apoptotic cell clearance from the inflamed airways. Interestingly, not only efferocytosis but also engulfment of bacteria by airway macrophages is impaired in asthma [105]. This finding indicates that alterations in the intracellular molecular machinery regulating phagocytosis rather than changes in expression of receptors recognising apoptotic cells might be responsible for the observed phagocytosis defects.

Finally, while previous studies have solely focused on efferocytosis by airway macrophages, mounting evidence suggests that bronchial epithelial cells might be equally important in clearing apoptotic cells from the inflamed airways. Even though epithelial cells are not professional phagocytes, they efficiently engulf apoptotic eosinophils, but not neutrophils [94, 106]. Animal studies demonstrate a critical role for airway epithelial cells in the removal of apoptotic cells from the inflamed lung and, as a consequence, control of inflammatory responses in the murine model of allergic airway hyper-responsiveness [68]. Future studies in humans are therefore necessary to characterise the relative contributions of airway macrophages and bronchial epithelial cells to defects in apoptotic cell removal in asthma and to identify therapeutic strategies which could restore and/or promote the efferocytic function of both cell types, as well as activate anti-inflammatory transcriptional programmes associated with apoptotic cell recognition.

### Cystic fibrosis

Cystic fibrosis (CF) is a heritable disorder caused by mutation in the CF transmembrane conductance regulator (CFTR) and characterised by severe pulmonary manifestations. Impaired mucociliary clearance in CF patients prevents elimination of bacteria from the lung, leading to persistent neutrophilic inflammation and progressive, irreversible damage of the airways [107]. Similar to other lung diseases associated with chronic inflammation, both accumulation of apoptotic cells in the airways and reduced numbers of phagocytic bodies within sputum macrophages are observed in patients with CF compared to control patients with chronic bronchitis [25]. Mechanistically, degranulation products of immune cells are partly responsible for efferocytosis defects in CF as neutrophil elastase present in the airway fluid of CF patients selectively cleaves PtdSer recognition receptors and suppresses apoptotic cell removal by airway macrophages [25]. Other potential mechanisms contributing to impaired apoptotic cell clearance in CF involve the release of HMGB1 which, similar to COPD, is elevated in sputum samples from CF patients [108] and effects of bacterial products on airway macrophages. *Pseudomonas aeruginosa* infections are common in CF and represent an important cause of mortality in CF lung manifestations. In vitro, *P. aeruginosa* toxic metabolite pyocyanin and the polysaccharide alginate inhibit apoptotic cell uptake by macrophages [109, 110], though the relevance of this mechanism in patients has yet to be demonstrated. Interestingly, animal studies indicate that airway epithelial cells in CF might also be deficient in phagocytic functions. 

While defective efferocytosis by airway macrophages in CF patients is a consequence of the ongoing inflammatory response and/or microbial infection, impaired apoptotic cell uptake by epithelial cells might be directly related to the lack of CFTR expression. CFTR-deficient epithelial cells express significantly increased levels of RhoA, which is a negative regulator of efferoctyosis, and RhoA inhibition restores their phagocytic function [111]. It remains to be determined if a similar mechanism regulates bronchial epithelial cell efferocytosis in patients with CF.

### Pulmonary fibrosis

Elevated levels of apoptotic cells and reduced frequencies of phagocytic bodies within bronchoalveolar lavage macrophages have also been reported in patients with idiopathic pulmonary fibrosis (IPF) [112]. IPF is an interstitial lung disease characterised by epithelial injury that is followed by aberrant alveolar wound repair and scar formation, which ultimately lead to respiratory failure and death. IPF is frequently accompanied by chronic neutrophilic inflammation [113]. Interestingly, intratracheal instillation of apoptotic cells ameliorates fibrosis and inflammation in bleomycin-induced lung injury in mice [58, 114], indicating that signalling triggered by apoptotic cell recognition may play a protective role in lung diseases associated with dysregulated healing processes. The anti-fibrotic effects of apoptotic cells in this model are dependent on the induction of PPARγ expression in airway macrophages and increased production of hepatocyte growth factor (HGF), which plays a key role in alveolar epithelial repair upon lung injury [58, 114]. These observations suggest that defects in efferocytosis in IPF patients may be responsible not only for inefficient clearance of apoptotic cells but also for diminished production of factors that support tissue repair without fibrosis.

Collectively, the data from patients with asthma, COPD, CF and pulmonary fibrosis indicate that defective apoptotic cell clearance in lung diseases is not specific for individual diagnoses but rather represents a general hallmark of chronic inflammation. Although several mechanisms contributing to these defects have been proposed, it remains to be verified whether impairment of efferocytosis might be a direct cause of chronic inflammation, or is a consequence to the ongoing inflammatory processes that contributes to chronicity and prevents resolution. The latter model is supported by the observation that mice lacking the TAM receptor Axl do not develop spontaneous lung inflammation despite defects in apoptotic cell uptake by airway macrophages [2], but more detailed analyses of regulation and function of PtdSer recognition receptors in the human lung are required. Finally, because recognition of apoptotic cells by PtdSer-recognising receptors activates downstream signalling pathways even without engulfment [46], future studies are needed to verify whether activation of transcriptional programmes triggered by recognition of apoptotic cells is also altered in chronic lung diseases and, if so, how they can be manipulated in the clinic by specific modulators of PtdSer recognition receptors.

## Efferocytosis in microbial infection of the lung

While in sterile inflammation recognition of apoptotic cells by the immune system typically results in suppression of the ongoing inflammatory response, the complexity of this process and its biological effects greatly increase in the context of microbial infections. First, some enveloped viruses use PtdSer receptors to promote their entry into the host cells and facilitate infection and immune evasion [115]. The interaction between PtdSer exposed on the surface of the viral envelope and members of the TAM and TIM receptor families expressed on the target cell enables proximity to specific entry receptors and enhances engulfment of the virus [116] (Fig. 1c). The relevance of this process, called ‘apoptotic mimicry’, has recently been confirmed for a broad range of virions, including Ebola and dengue viruses [117, 118]. Thus far, however, little is known about potential roles of this entry mechanism in pulmonary viral infections. The observation that the kinetics of influenza H1N1 virus clearance was unaffected in Axl-deficient mice indirectly indicates that Axl is not important for control of this infection [2], though Axl-H1N1 interaction has not been formally tested. Similarly, although TIM1 promotes internalisation and replication of several enveloped viruses, TIM1-mediated entry does not lead to a productive infection by influenza H7N1 and severe acute respiratory syndrome (SARS) coronavirus [117], arguing against an important role of apoptotic mimicry in viral lung diseases.

Second, many pathogens survive intracellularly, and phagocytosis of infected host cells undergoing apoptosis has diverse consequences for pathogen survival and immune response of the host [119]. One of the main strategies of the immune system to control intracellular infections is through induction of apoptosis of infected cells, which are then engulfed and destroyed together with the pathogen by phagocytes (Fig. 1d). However, in some cases, efferocytosis of infected cells is used by intracellular pathogens, such as the parasite *Leishmania major*, to evade the immune response and gain entry into the new cellular host [119]. Among pathogens important in respiratory diseases, the role of efferocytosis has been most thoroughly studied in the context of *Mycobacterium tuberculosis* infections. *M. tuberculosis* infects macrophages and induces necrosis of the host cell to avoid clearance by the immune system and disseminate [120]. Interestingly, *M. tuberculosis*-infected mouse macrophages which die by apoptosis are rapidly efferocytosed by uninfected macrophages, leading to bacterial killing and elimination [121]. This bactericidal effect is dependent on efferocytosis, as the uptake of naked *M. tuberculosis* does not allow for lysosome recruitment to the bacteria-containing phagosome, and inhibition of apoptotic cell uptake with a TIM-4-blocking antibody prevents bacterial control in vitro and increases bacterial burden in the lungs in vivo [121]. The observation that bactericidal activity of macrophages infected with *Streptococcus pneumoniae* is dependent on induction of macrophage apoptosis suggests that a similar mechanism might be involved in controlling infection with this bacterium [122], though the involvement of efferocytosis in this process has not been formally proven. Engulfment of macrophage-derived apoptotic vesicles also plays a critical, though indirect, role in the adaptive immune response against *M. tuberculosis*: annexin A1-dependent uptake of apoptotic cells by dendritic cells is required for cross-presentation and generation of *M. tuberculosis*-specific CD8 T cell response and bacterial clearance from the lung [123].

The antimicrobial role of apoptotic cell clearance in pulmonary infections is not restricted to bacteria as the influenza A virus induces apoptosis of epithelial cells upon infection and engulfment of influenza A-infected cells by macrophages is associated with reduction of viral titres [124]. On the other hand, infection of macrophages with *Francisella novicida*, a member of the *Francisellaceae* family of intracellular bacteria which cause pulmonary inflammation associated with necrotic infiltrates in the lung, reduces their efferocytic function, potentially contributing to the accumulation of necrotic cell debris and exacerbation of disease [125].

Taken together, these studies suggest that efferocytosis is not only a constitutive function of macrophages required for maintaining immune homeostasis in health and during inflammatory response but also an important antimicrobial effector mechanism. However, it has to be noted that apart from bactericidal activity against specific pathogens, the anti-inflammatory signal associated with apoptotic cell uptake by phagocytes might prevent the mounting of an efficient immune response in the context of other lung infections. Prior exposure of mouse airway macrophages to apoptotic cells results in suppression of FcR-mediated phagocytosis and killing of bacteria, and intrapulmonary administration of apoptotic cells causes significant impairment of *S. pneumoniae* clearance from the infected lung [56]. Suppression of antimicrobial responses of airway macrophages is also augmented by glucocorticoids, which promote efferocytosis, and treatment of mice with apoptotic cells in the presence of glucocorticoids is associated with elevated bacterial burden in the lungs [126]. Even though validation of these observations in human systems is necessary, they clearly indicate that efferocytosis plays a dual role in lung infections: while efficient apoptotic cell uptake is required for resolution of the inflammatory response and elimination of certain intracellular pathogens, the anti-inflammatory programmes activated upon prolonged exposure to apoptotic cells might increase susceptibility to secondary infections and infection-related exacerbations of chronic inflammatory lung diseases.

## Targeting defective apoptotic cell clearance in lung diseases

In light of the importance of effercotysis in resolution of inflammation and the reported defects in apoptotic cell removal in chronic inflammatory lung diseases, it is not surprising that evaluation of potential therapeutic strategies aimed at enhancing apoptotic cell uptake gained a lot of attention in recent years [72, 73]. Strikingly, glucocorticoids, which are the most commonly used class of drugs in the treatment of asthma and COPD, increase apoptotic cell engulfment by macrophages in vitro [127] and restore the efferocytic function of airway macrophages from patients with severe asthma [97]. Upregulation of the TAM receptor MerTK [128, 129] and downregulation of SIRPα [130] might be responsible for the pro-efferocytic activity of glucocorticoids in macrophages. However, it remains unknown whether promotion of apoptotic cell clearance significantly contributes to the anti-inflammatory effects of glucocorticoids in patients with lung diseases. More systematic analyses of apoptotic cell accumulation and efferocytic functions of airway macrophages before and after glucocorticoid therapy in responders and non-responders are therefore necessary to address this question.

Some classes of medications widely used in the clinic for other indications also promote efferocytosis. The antibiotic azithromycin increases phagocytosis of apoptotic cells by human airway macrophages in vitro [21, 131], and significant improvement of the efferocytic function of airway macrophages from COPD patients after oral treatment with azithromycin has been reported [28]. In light of recent evidence that azithromycin reduces the frequency of exacerbations in COPD patients [132], it is tempting to speculate that at least part of the immunomodulatory activities of macrolide antibiotics can be attributed to their effects on apoptotic cell clearance. Similarly, PPARγ agonists, which are used as insulin sensitizers in diabetes mellitus, but also display broad anti-inflammatory effects, promote efferocytosis by airway macrophages in vitro and ameliorate disease symptoms in animal models of pulmonary inflammation [133, 134]. Enhancement of apoptotic cell engulfment is also observed after treatment of macrophages from COPD patients with simvastatin [135], a member of the statin family of cholesterol-lowering drugs, and retrospective studies suggested that statins might reduce the risk of COPD exacerbations and mortality [136, 137]. More recently however, in a large randomised clinical trial, simvastatin treatment had no effect on exacerbation rates in COPD patients [138], though the efferocytic function of airway macrophages was not analysed in this study. It remains an open question if patients with COPD can be stratified to identify a subgroup of patients that responds to statin treatment and whether this is related to effects on apoptotic cell clearance.

Finally, the observations that aberrant expression and activity of the TAM receptor Axl has an oncogenic function in haematological and epithelial malignancies triggered the interest in targeting the activity of PtdSer recognition receptors, and a small molecule inhibitor of Axl is currently in phase I clinical trials [139]. However, preclinical studies indicate that pharmacological modulators of PtdSer recognition receptors might have a significant impact on the immune system, especially in the context of lung immunopathology. In light of the critical role of Axl in resolution of lung inflammation upon influenza infection [2], any attempts to target Axl activity systemically should proceed with caution due to potential adverse events related to exaggerated inflammatory responses to respiratory infections. Similar to Axl, MerTK signalling is also required for silencing of lung inflammation: inhibition of MerTK proteolytic cleavage by the ADAM17 inhibitor TAPI-0 restores MerTK expression and attenuates inflammation during LPS-induced lung injury [140], whereas administration of a MerTK blocking antibody has the opposite effect [141]. These results suggest that activating antibodies or compounds which prevent shedding of TAM receptors could be beneficial in the context chronic lung diseases. Indeed, TAM receptors can be activated independently of apoptotic cell engulfment by specific antibodies [46, 142], though their effects have not been tested in models of lung inflammation. In that regard, it is noteworthy that activation of TAM receptors leads to shedding of their extracellular domains and soluble forms of TAM receptors can act as decoy receptors and suppress apoptotic cell engulfment by macrophages [143]. Although alterations in the levels of soluble TAM receptors are noted in several pathologies [144, 145], their physiological role is still poorly understood. It remains unknown if administration of antibodies targeting TAM receptors would cause their sequestration and what consequences it would have for the immune homeostasis of the lung.

## Conclusions

Since the initial discovery of defects in apoptotic cell clearance in asthma and COPD, great progress has been made in our understanding of the molecular mechanisms of efferocytosis and several new processes through which efferocytosis modulates host immune responses have been characterised (Fig. 1). Consequently, new questions have emerged regarding the role of apoptotic cell removal in lung homeostasis and the most important of them are listed in Box 1. Future studies addressing these questions, especially attempts to therapeutically manipulate efferocytosis in the clinic, should be designed in the context of the multiple roles of apoptotic cell phagocytosis in resolution of inflammation and microbial infections.

Box 1. Future questions in apoptotic cell removal in the airspaces

**Table:** 

• Does efferocytosis polarise airway macrophages to an M2 phenotype or are M2 polarised airway macrophages better at efferocytosis?
• What is the impact of the tissue microenvironment on apoptotic cell recognition receptor repertoires and outcome of their ligation?
• Is homeostatic apoptotic cell clearance different in requirements and consequences to clearance of cells during inflammation?
• What is the impact of the altered lung environment in asthma, COPD and CF on apoptotic cell clearance?
• Does cooperation between different apoptotic cell clearance receptors introduce heterogeneity in outcome?
• Under what circumstances is efferocytosis by non-professional phagocytes important?
• To what extent do PtdSer-expressing pathogens sculpt immunity?
• Should there be an effort to develop therapeutics that manipulate the efferocytic pathway?
